# Long-term outcomes for children with disability and severe acute malnutrition in Malawi

**DOI:** 10.1136/bmjgh-2020-002613

**Published:** 2020-10-07

**Authors:** Natasha Lelijveld, Nora Groce, Seema Patel, Theresa Nnensa, Emmanuel Chimwezi, Melissa Gladstone, Macpherson Mallewa, Jonathan Wells, Andrew Seal, Marko Kerac

**Affiliations:** 1 Institute for Global Health, University College London, London, UK; 2 Clinical Research Programme, Malawi-Liverpool Wellcome Trust, Blantyre, Malawi; 3 Department of Epidemiology and Health Care, UCL International Disability Research Centre, London, UK; 4 Institute of Translational Medicine, University of Liverpool, Liverpool, UK; 5 Department of Paediatrics and Child Health, University of Malawi College of Medicine, Blantyre, Southern Region, Malawi; 6 Childhood Nutrition Research Centre, Institute of Child Health, University, London, UK; 7 Department of Population Health, London School of Hygiene and Tropical Medicine, London, UK

**Keywords:** nutrition, child health

## Abstract

**Introduction:**

Severe acute malnutrition (SAM) and disability are major global health issues. Although they can cause and influence each other, data on their co-existence are sparse. We aimed to describe the prevalence and patterns of disability among a cohort of children with SAM.

**Methods:**

A longitudinal cohort study in Malawi followed SAM survivors up to 7 years postdischarge. Clinical and anthropometric profiles were compared with sibling and community controls. Disability at original admission was identified clinically; at 7-year follow-up a standardised screening tool called ‘the Washington Group Questionnaire’ was used.

**Results:**

60/938 (6.4%) of admissions to SAM treatment had clinically obvious disability at admission. Post-treatment mortality was high, with only 11/60 (18%) surviving till 7-year follow-up. SAM children with a disability at admission had 6.99 (95% CI 3.49 to 14.02; p<0.001) greater risk of dying compared with children without disability. They were also older, less likely to be HIV positive or have oedema and more severely malnourished. Long-term survivors were more stunted, had less catch-up growth, smaller head circumference, weaker hand grip strength and poorer school achievement than non-disabled survivors.

The Washington Group Questionnaire confirmed disability in all who had been identified clinically, and identified many who had not been previously flagged.

**Conclusion:**

Disability is common among children affected by SAM. Those with disability-associated SAM have greatly increased risk of dying even if they survive the initial episode of malnutrition. Survivors have poorer growth, physical strength and school achievement. To enable all children to survive and thrive post-SAM, it is vital to focus more on those with disabilities. SAM treatment programmes should consider using not just clinical assessment but structured assessments to better identify at-risk individuals as well as understand the population of children for which they are developing services.

**Key questions**

**What is already known?**

- Severe acute malnutrition (SAM) and disability are important global health issues.
- Although each can cause and influence the other, data on their co-existence are sparse.

**What are the new findings?**

- Disability is common among children affected by SAM, especially cerebral palsy and developmental delay.
- Children with SAM and underlying disability have greatly increased risk of death—almost 7 times greater risk compared with those who had SAM but no underlying disability.
- Clinical identification alone fails to identify many children with disability.

**What do the new findings imply?**

- To help children survive and thrive post-SAM, it is vital that those with underlying disability are identified and supported.
- SAM treatment programmes should consider using more structured assessments, such as screening tools, to better understand the population of children they are serving: clinical assessment alone is likely to be inadequate.
- Our findings concur with emerging evidence suggesting that SAM programmes need to tailor treatment towards risk rather than severity of anthropometry only.
- As we consider the future of treatment programmes, it is imperative that identification and support for disability is included.

## Introduction

There is increasing international awareness of the links between malnutrition and disability: both are major global health problems, and both are key human rights concerns. Severe acute malnutrition (SAM) affects >14 million children aged under 5 years worldwide.^1^ Moderate or severe disability affects around 93 million children aged 0–14 years worldwide.^2^ However, links between these two global health problems in low-income settings are poorly understood and seldom considered by those working in the two respective fields.

Disability can be both caused by SAM and can result in a greater risk of SAM.^3^ Despite this knowledge, there is little data on the prevalence of children with both conditions in low-resource settings, and the diagnosis and management of each condition often does not consider the other. This is compounded by the fact that many SAM research studies exclude children with disabilities from their sample or analysis.^4^

Our study cohort (the ‘Pronut’ cohort) were originally admitted to the child malnutrition ward in the Queen Elizabeth Central Hospital Blantyre, Malawi, for treatment of SAM in 2006/2007.^5^ All admissions, including those with disabilities were enrolled. The cohort was followed-up at 1 year postdischarge (FuSAM study, follow-up post-SAM) and again at 7 years postdischarge (ChroSAM study, chronic disease outcomes post-SAM).^6 7^ Since the advances in the treatment of SAM as well as a number of other important child health interventions, more children globally are surviving to see their fifth birthday. This has resulted in a considerable gap in our knowledge of both the short-term and long-term effects for SAM survivors.^8 9^ Even less is known about outcomes for children with disabilities due to challenges in diagnosis, measurements, and follow-up.

To fill some of the above evidence gaps, our aim was to use our SAM cohort from Malawi to describe the prevalence, characteristics and outcomes of disability among children with SAM. Objectives were to:

- describe the short-term and long-term post-SAM outcomes among children with disability compared with those with no underlying disability;
- explore two different methods of identifying disability among children with SAM: clinical assessment vs structured assessment using a validated tool.^10^

Understanding these basic issues will help inform future approaches and interventions for diagnosis, immediate management and long-term management of children with SAM who have a disability. It is also key to advocating for better malnutrition prevention strategies within this group.

## Methods

This was a secondary analysis arising from two longitudinal cohort studies prospectively following up survivors who had been treated for SAM, 1 and 7 years postdischarge from a hospital in Blantyre, Malawi. Non-SAM sibling and age-matched/sex-matched community controls were also recruited for at the 7-year follow-up.

### Study setting and subjects

Full details of the cohort, as well as additional methods and results on other outcomes are described elsewhere.^5–7 11–14^ In brief, the original cohort included all patients admitted to the nutrition ward of Queen Elizabeth Central Hospital, Blantyre, Malawi, from 12 July 2006 to 9 March 2007 (1024 children). At the time, Community Management of Acute Malnutrition was not available so all children were admitted to initial inpatient care but once clinically stable were followed up at fortnightly outpatient clinics. All admissions, including those with disabilities, were enrolled. The cohort was followed up at 1-year postdischarge and again at 7 years postdischarge.^5–7^ The median age at admission was 21.5 months (IQR 15–32 months). Results of survival and anthropometry analysis at baseline and the 1-year follow-up have been described previously.^5 6^ Sibling controls were those closest in age to the SAM survivor (‘case child’), between the ages of 6 and 15.9 years; community controls were those living in the same community, of the same sex, and within 12 months of age of the case child, randomly selected by spinning a bottle at the case child’s home to select a random direction, then enquiring door-to-door to find the first eligible child. Children who had ever been treated for acute malnutrition were excluded from the control group. Informed written consent was obtained from the child’s parent or guardian; assent from the children themselves.

### Variables

At admission and each follow-up, we collected anthropometric data (height, weight, mid-upper arm circumference) using duplicate measures by two data collectors, following the method recommended by WHO.^15^ Weight-for-height (WHZ), weight-for-age (WAZ), height-for-age (HAZ) and body mass index (BMI)-for-age (BAZ) scores were calculated using WHO growth standards.^16^ At the 7-year follow-up, we also measured waist circumference and hip circumference, and hand grip strength using a Takei Grip-D device (Takei, Niigata, Japan). School grade achieved was used as a measure of educational attainment since children in Malawi do not move to the next grade until they pass, irrespective of their age. HIV status was established from results in health passports; or if unknown, an HIV test was offered by a trained counsellor. If a child had died, this had to be reported by the main caregiver. If the main caregiver could not be located, this was recorded as loss-to-follow-up. Whole-body bioelectrical impedance (Z, ohms) was measured with the child supine using a Quadscan 4000 device (Bodystat, Douglas, Isle of Man). The impedance index (HT2/Z) was calculated as an index of fat-free mass.

At original admission, a clinician (doctor or clinical offer) noted presence of any clinically obvious disability (eg, cerebral palsy (CP), Down syndrome). The diagnosis was confirmed by a senior paediatrician but details and severity were not further described at the time. At the 7-year follow-up, we used a formal assessment tool, the ‘Washington Group’ questions (questions 1–6 from the Short Set with additional questions 7–14 from the Extended Set (parent reported)).^10^ These explored physical and behaviour difficulties experienced by children, as reported by their caregiver. We categorised questions 1–4 (difficulty seeing, hearing, walking or self-care) as ‘physical difficulties’, and the rest of the questions (5-14; difficulty understanding, being understood, learning, remembering, worrying, controlling behaviour, completing tasks, change of routine, getting along with other children and difficulty playing) as ‘behaviour and learning difficulties’. The questionnaire was administered by trained study nurses. The tool, a product of a UN Statistics working group, (The Washington Group on Disability Statistics) has been widely validated in low-income, middle-income and high-income countries. It is currently used by National Statistics Offices in over 80 countries and has been identified as the primary methodology for disability statistics collection related to the Sustainable Development Goals, as well as being widely used in research studies (www.washingtongroup.com).

### Sample size and analysis

Sample size was predetermined by the cohort size and survival rates. Controls were more difficult to recruit throughout the study in comparison to cases because they had no previous personal connection with the study team and were restricted to the number of eligible children in the family and community.

We used Stata V.14 to perform all analyses (StataCorp, College Station, Texas, USA). We included age, sex and HIV status as a priori potential confounders in all multivariable regressions. We used multivariable logistic regression and Kaplan-Meier survival curves to explore differences in survival between those with a clinically obvious disability and those without. We also used multivariable logistic regression for categorical outcomes and multivariable linear regression for continuous outcomes, to assess differences in admission characteristics between those with a clinically obvious disability and those without; differences in response to Washington Group questions between SAM survivors and control groups and differences in outcomes within the SAM survivors at 7 years postdischarge for children with versus without a clinically obvious disability, as well as those who had physical versus no physical difficulties based on the Washington Group questions, and those with behaviour versus no behaviour difficulties.

## Results

According to the original clinical assessment at admission for SAM treatment, 60/938 (6.4%) had an obvious disability. Three hundred fifty-two of the cohort were found to be alive at 1-year postdischarge, and 314 at 7 years postdischarge. Of those with a clinically obvious disability:

- 18/60 (30%) were still known to be alive at 1-year follow-up, hence 18/352 (5.1%) of survivors had a disability at 1-year follow-up;
- 11/60 (18%) were still known to be alive at 7-year follow-up, hence 11/314 (3.5%) survivors have a disability at 7-year follow-up (42 had died and 7 were lost-to-follow up).

Table 1 describes the types of disabilities identified at admission, and details how many individuals progressed to each follow-up stage. Most children had CP or ‘developmental delay’.

**Table 1:** Description of types of disability identified clinically at admission for SAM treatment

Type of disability (as recorded in clinical notes)	Admission (n=60)	1-year follow-up (n=18)	7-year follow-up (n=11)
Cerebral palsy	34	10	6
Down syndrome	1	1	1
Developmental delay	10	2	1
Musculoskeletal condition	1	1	1
Right hemiplegia	1	1	0
Hydrocephalus	3	0	0
Pierre Robin syndrome	1	0	0
Talipes (clubfoot)	1	0	0
Mild physical difficulty walking	1	0	0
Unspecified disability	7	3	2

### Survival

**Figure 1:**
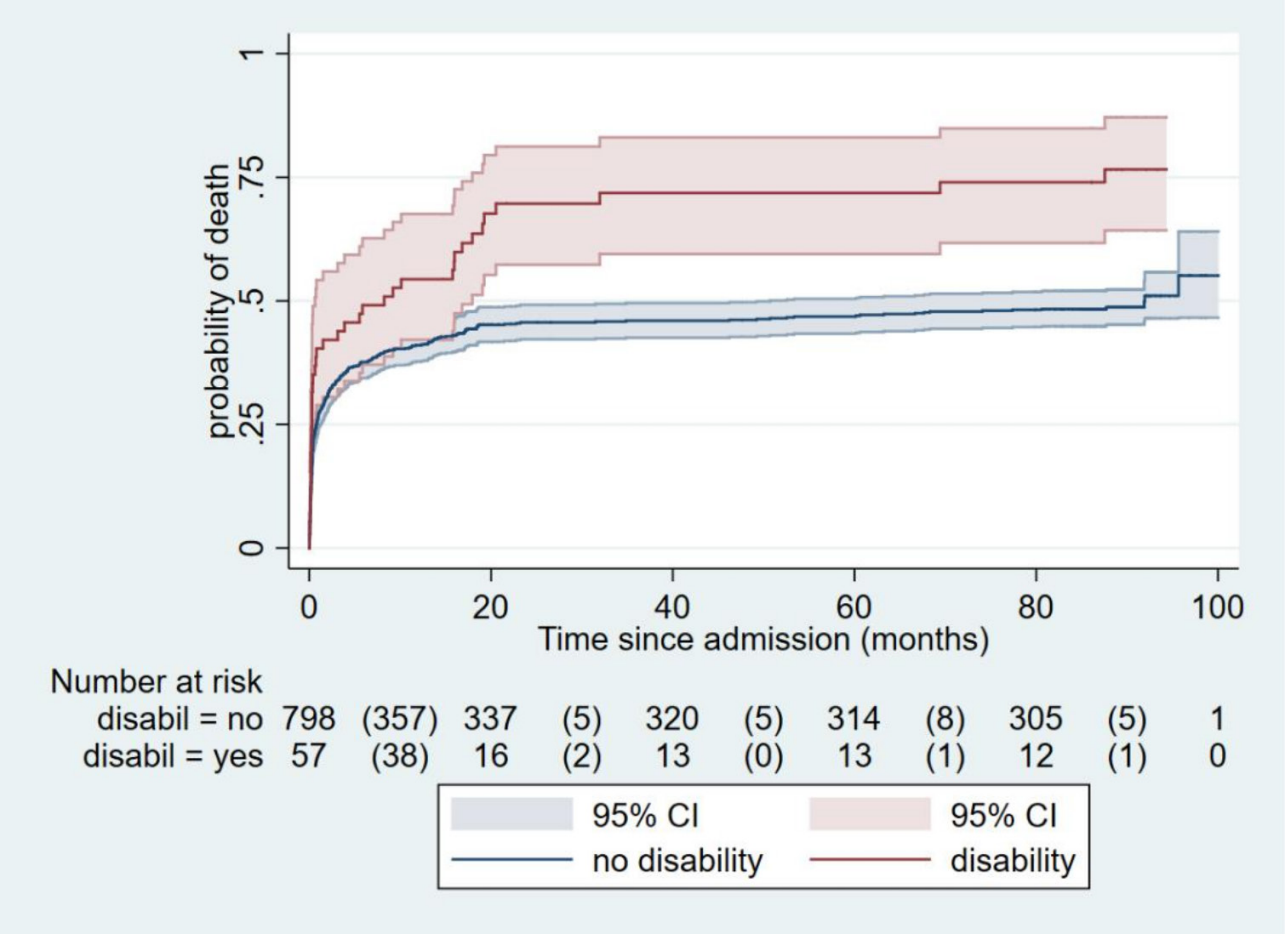
Kaplan-Meier survival curves, for those with disability versus no disability.

Children with a (clinically identified) disability at admission had a much greater risk of dying by 7-year postdischarge compared with those admitted without a disability (figure 1) (unadjusted relative risk (RR) 3.03 (95% CI 1.65 to 5.56; p<0.001); RR adjusted for age, sex and HIV status 6.99 (95% CI 3.49 to 14.02; p<0.001).

At the 1-year follow-up, we found a high mortality rate (42%) for children with SAM, both during treatment and within 1 year of discharge from treatment. Two groups of children were particularly vulnerable: those with HIV, who made up 274 (62%) of the children who died and those with disabilities who were HIV negative (n=15 (4%)), who were 2.8 times more likely to die during follow-up than children without disabilities (95% CI 1.4 to 5.3).^6^ By year 7, children with disabilities were 6.99 times more likely to die compared with non-disabled patients with SAM. As noted below, we also found long-term adverse effects on growth, body composition, muscle strength and school achievement, for SAM survivors compared with sibling and community controls.^7^

Although the sample size is small, it is important to point out that in addition to overall increased mortality rates, children with some types of disability were at particular risk. By year 7, 82% of children with CP had died.

### Differences between SAM admissions with and without a clinically obvious disability

Children with a clinically obvious disability were older on average and had a slightly higher proportion of males than children without a disability at admission (table 2). Those with a disability at admission were also less likely to be HIV positive and less likely to have oedema than children without a disability. At admission they also had a 1.31 lower mean WHZ score, 1.27 lower WAZ score and 0.78 lower HAZ score (table 2 and online supplemental annex table 1). Adjusted OR for being HIV positive if a child had a disability, compared with no disability, was 0.34 (95% CI 0.18 to 0.65; p=0.001), and for having nutritional oedema at admission is 0.44 (95% CI 0.22 to 0.88; p=0.02).

10.1136/bmjgh-2020-002613.supp1 Supplementary data

**Table 2:** Description of children with and without a clinically obvious disability in the sample

At original admission	Clinically obvious disability n=60	No obvious disability n=878
Age (months) (median, IQR)	29 (14–55)	21 (15–31)
Males	38 (63.3%)	457 (52.3%)
Known HIV positive	13 (21.6%)	407 (46.4%)
Oedema	39 (65.0%)	618 (70.4%)
Weight (kg) (minimum on ward) (mean (SD))	7.5 (3.4)	7.4 (2.6)
Length (cm) (mean (SD))	77.7 (13.9)	76.2 (11.6)
MUAC (mean (SD))	11.5 (2.5)	11.6 (2.0)
WHZ (mean (SD))	−3.55 (1.99)	−2.70 (1.82)
WAZ (mean (SD))	−4.58 (1.55)	−3.66 (1.61)
HAZ (mean (SD))	−4.11 (1.66)	−3.37 (1.46)

At 7-year follow-up	Clinically obvious disability n=11	No obvious disability n=303
Age (months) (median, IQR)	142 (124–151)	111 (103–122)
Males	8 (73%)	161 (53%)
Known HIV positive	2 (18.2%)	86 (28.4%)
Weight (kg) (mean (SD))	26.0 (9.1)	23.9 (4.9)
Height (cm) (mean (SD))	124.3 (17.0)	125.0 (9.0)
MUAC (mean (SD))	179.6 (40.6)	172.1 (19.0)
BAZ (mean (SD))	−1.55 (0.41)	−0.85 (0.94)
HAZ	−3.40 (2.57)	−1.80 (1.17)
HAZ gain since admission	−0.33 (1.26)	1.42 (1.42)
Waist circumference	56.4 (7.2)	56.2 (4.2)
Hip circumference	63.6 (10.8)	62.3 (5.7)
Head circumference	51.1 (3.5)	51.8 (2.0)
Hand grip strength	9.62 (6.6)	12.7 (6.4)
Impedance index (HT2/Z)	24.4 (8.8)	21.3 (4.9)
School grade achieved	1.0 (1.8)	2.5 (1.2)

WAZ and WHZ at 7-year follow-up is not presented as children are too old for these reference (WHZ is only for children aged <5 years, WAZ only for children aged <11 years) (only 1 child with a disability was aged <11 years by 7-year follow-up).

BAZ, BMI-for-age z-score; BMI, body mass index; HAZ, height-for-age z-score; MUAC, mid-upper arm circumference; WAZ, weight-for-age z-score; WHZ, weight-for-height z-score.

At 7-year follow-up, those admitted with a clinically obvious disability had a significantly lower HAZ, HAZ gain since admission, waist circumference, hip circumference, head circumference, hand grip strength and school achievement at 7-year postdischarge than those who were not diagnosed with a clinically obvious disability at admission (online supplemental annex figure 1 and table 2). As the growth references for WAZ is only applicable to children up to 11 years of age, and the SAM survivors with a disability tend to be older than those without regression was not conducted for WAZ. The small sample size of the SAM survivors with disabilities is also likely affecting the statistical significance of differences, since SAM survivors with a disability also have a lower mean BAZ compared with those without disabilities, but it is not statistically different.

### Overlap between those diagnosed as having a clinically obvious disability and reported difficulties using the Washington Group questions

We found that all of those identified with a clinically obvious disability at admission were reported to have ‘a lot or more’ difficulty by at least one of the Washington Group questions at 7 years post-SAM (table 3). Only one of these clinically identified children did not have a lot of physical difficulty, but did identify as having significant learning and behavioural difficulties.

**Table 3:** Differences between disability diagnosis at admission and ‘a lot of difficulty’ identified by Washington Questions* at 7 years postdischarge (SAM survivors only)

	Identified as having a disability at admission (n=11 survivors)	Not identified as having disability at admission (n=297 survivors)
Reported ‘a lot’ of physical difficulty at follow-up	10 (91%)	19 (6.4%)
Reported ‘a lot’ of learning/behaviour difficulties at follow-up	10 (91%)	90 (30.3%)
Reported ‘a lot’ of any difficulty at follow-up	11 (100%)	96 (32.3%)

*Options for responses to the questionnaire are: ‘no difficulty’, ‘some difficulty’, ‘a lot of difficulty’ and ‘cannot do at all’; these results show any children whose caregiver reported ‘a lot of difficulty’ or ‘cannot do at all’.

In contrast, many children without clinically obvious disability at original admission were identified as having problems at 7 years. This was particularly marked for learning/behavioural problems, with 30% (90/297) having a difficulty at 7 years that was not obvious at baseline. There was also increased identification of children not previously clinically identified as having physical difficulties, with an additional 6% (19/297) of children reporting ‘a lot of difficulty’ with one or more physical activities at 7 years.

### Prevalence of difficulties in survivors and controls using the Washington Group questions

**Figure 2:**
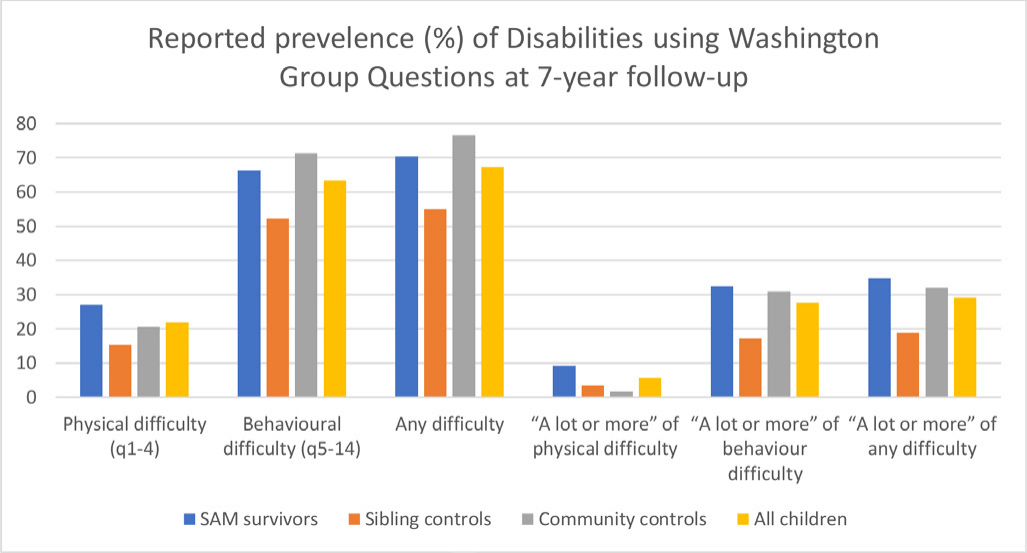
Bar chart showing prevalence of disabilities based on Washington Group questions at 7-year follow-up. SAM, severe acute malnutrition.

Children reported having ‘at least some’ physical difficulty ranged from 0.6% of community controls (walking) to 16.6% of SAM survivors (hearing) (online supplemental annex table 2). Of the behavioural difficulties, the highest prevalence was for ‘difficulty remembering’ among the SAM survivors (32.6%) and the lowest prevalence was for ‘difficulty being understood’ among the sibling controls (2.4%). Figure 2 (and online supplemental annex table 3) presents a summary of prevalence for ‘any physical difficulty’, ‘any behavioural difficulty’ and ‘any difficulty’, as well as the same categories for those who were categorised as ‘a lot of difficulty’ or greater.

**Are there differences in responses to the Washington Group questions for SAM survivors compared with controls?**

Crude analysis shows that sibling controls had significantly lower odds of having a behavioural difficulty, any difficulty, a lot of physical difficulty, a lot of learning/behaviour difficulty and a lot of any difficulty, than SAM survivors (after adjusting for age, sex and HIV) (table 4). Community controls had similar odds of having difficulties compared with SAM survivors, except for ‘a lot of physical difficulty’ where odds were significantly lower for community controls compared with SAM survivors.

**Table 4:** Associations between summary of difficulties on Washington Group disability questions at follow-up and surviving SAM

Type of disability	Unadjusted				Adjusted			
	Sibling controls vs SAM survivors OR (95% CI)	P value	Community controls vs SAM survivors OR (95% CI)	P value	Sibling controls vs SAM survivors OR (95% CI)	P value	Community controls vs SAM survivors OR (95% CI)	P value
Any physical difficulty (question 1–4)	0.49 (0.31 to 0.76)	**0.002**	0.70 (0.45 to 1.09)	0.11	0.66 (0.39 to 1.10)	0.11	0.89 (0.53 to 1.52)	0.68
Any learning/behaviour difficulty (5–14)	0.56 (0.39 to 0.79)	**0.001**	1.27 (0.85 to 1.91)	0.24	0.62 (0.41 to 0.93)	**0.02**	1.33 (0.85 to 2.11)	0.22
Any difficulty	0.51 (0.36 to 0.74)	**<0.001**	1.38 (0.90 to 2.10)	0.14	0.59 (0.36 to 0.89)	**0.01**	1.48 (0.92 to 2.39)	0.11
‘A lot’ of physical difficulty	0.34 (0.15 to 0.79)	**0.01**	0.17 (0.05 to 0.57)	**0.004**	0.30 (0.11 to 0.85)	**0.02**	0.23 (0.06 0.86)	**0.03**
‘A lot’ of learning/behaviour difficulty	0.43 (0.28 to 0.66)	**<0.001**	0.93 (0.62 to 1.38)	0.71	0.50 (0.31 to 0.80)	**0.004**	1.08 (0.68 to 1.72)	0.73
‘A lot’ of any difficulty	0.43 (0.28 to 0.66)	**<0.001**	0.89 (0.60 to 1.31)	0.54	0.51 (0.32 to 0.81)	**0.004**	1.07 (0.68 to 1.69)	0.77

Logistic regression, both unadjusted and adjusted for age, sex and HIV status. P values <0.05 are statistically significant (in bold).

SAM, severe acute malnutrition.

Applying multivariable logistic regression to adjust for age, sex and HIV status, we found that sibling controls have significantly reduced odds of difficulty hearing, understanding, being understood, learning, remembering and completing tasks, compared with SAM survivors (online supplemental annex table 4). None of the questions was significantly different when comparing SAM survivors with community controls. When applying the same analysis, but for ‘a lot or more’ difficulty to all of the questions, siblings had significantly reduced odds of difficulty hearing, understanding, remembering and controlling their behaviour. Community controls had significantly reduced odds of having difficulty hearing, and difficulty remembering. No community controls reported a lot of difficulty seeing or walking, compared with 5/314 and 10/314, respectively for SAM survivors.

### Other associations with Washington Group questions

In the whole sample, HIV-positive children were significantly more likely to have behavioural difficulties (after adjusting for SAM survival, age and sex=OR 1.75, 95% CI 1.05 to 2.93, p=0.03), physical difficulties (OR 2.46, 95% CI 1.48 to 4.09, p=0.001), and ‘any difficulty’ (OR 1.84, 95% CI 1.07 to 3.16, p=0.03), compared with HIV-negative children. Boys reportedly worried more often than girls (OR 1.56, 95% CI 1.07 to 2.27, p=0.02). There was no association between any of the difficulty questions and age.

For growth and other outcomes, the SAM survivors classified as having ‘a lot or more difficulty’ with any of the physical indicators in the Washington Group questions, had a significantly lower HAZ, WAZ, waist circumference and school achievement than those without a lot of physical difficulty. Only two outcomes were significantly different between those with ‘a lot of behavioural difficulty’ and those without: waist circumference and school achievement (table 5).

**Table 5:** Differences in outcomes for children with and without difficulties at 7 years postdischarge (identified by WGQ)

	A lot of any physical ‘difficulty’ (identified at 7-year follow-up by WGQ)			A lot of any learning/behavioural ‘difficulty’ (identified at 7-year follow-up by WGQ)		
	SAM survivors without difficulty n=274	SAM survivors with difficulty n=24	Difference (95% CI) p value	SAM survivors without difficulty n=206	SAM survivors with difficulty n=91	Difference (95% CI) p value
HAZ	−1.76 (1.15)	−2.46 (1.60)	−0.52 (−1.04 to 0.01) **0.05**	−1.77 (1.21)	−1.88 (1.18)	−0.09 (−0.37 to 0.19) 0.55
WAZ	−1.50 (0.92)	−2.24 (1.18)	−0.74 (−1.31 to –0.18) **0.01**	−1.51 (0.93)	−1.62 (1.00)	−0.12 (−0.40 to 0.16) 0.41
BAZ	−0.83 (0.94)	−1.15 (0.92)	−0.25 (−0.68 to 0.18) 0.25	−0.83 (0.96)	−0.91 (0.88)	−0.07 (−0.30 to 0.16) 0.55
MUAC	172.4 (18.6)	175.2 (27.5)	−2.6 (−9.9 to 4.7) 0.49	173.3 (19.4)	171.2 (19.5)	−3.02 (−7.21 to 1.17) 0.16
WAZ gain since admission	1.73 (1.46)	1.31 (1.40)	−0.20 (−1.00 to 0.60) 0.62	1.76 (1.50)	1.57 (1.36)	−0.16 (−0.56 to 0.23) 0.41
HAZ gain since admission	1.41 (1.28)	1.19 (1.54)	−0.001 (−0.63 to 0.63) 0.99	1.43 (1.27)	1.31 (1.37)	−0.08 (−0.41 to 0.25) 0.65
Waist circumference	56.3 (4.1)	56.5 (5.8)	−1.56 (−3.12 to –0.01) **0.04**	59.5 (4.1)	55.8 (4.5)	−0.99 (−1.90 to –0.10) **0.03**
Hip circumference	62.3 (5.7)	63.3 (7.2)	−1.01 (−3.07 to 1.06) 0.34	62.4 (5.7)	62.4 (6.0)	−0.34 (−1.49 to 0.80) 0.56
Head circumference	51.8 (2.1)	51.8 (2.1)	−0.71 (−1.56 to 0.15) 0.10	51.9 (2.1)	51.7 (1.9)	−0.33 (−0.82 to 0.16) 0.19
Hand grip strength	12.8 (6.4)	12.3 (5.2)	−2.09 (−4.79 to 0.60) 0.13	13.0 (7.0)	12.3 (4.1)	−0.85 (−2.36 to 0.66) 0.27
Impedance index (HT2/Z)	21.4 (4.8)	21.5 (6.4)	−1.90 (−3.87 to 0.07) 0.06	21.2 (4.8)	21.7 (5.2)	0.16 (−0.90 to 1.23) 0.76
School grade achieved	2.5 (1.3)	1.7 (1.6)	−0.96 (−1.44 to –0.48) **<0.001**	2.6 (1.2)	2.1 (1.3)	−0.44 (−0.72 to –0.17) **0.002**

Linear regression adjusted for age, sex and HIV status. We could not assess differences in WAZ for children with a clinically obvious disability as only one child has a valid WAZ since it can only be calculated for children aged <11 years. Although mean circumferences appear similar, a significant difference emerges after adjusting for age, as children with disabilities are significantly older than children without. P values <0.05 are statistically significant (in bold).

BAZ, BMI-for-age z-score; BMI, body mass index; HAZ, height-for-age z-score; MUAC, mid-upper arm circumference; SAM, severe acute malnutrition; WAZ, weight-for-age z-score; WGQs, Washington Group Questionnaire.

## Discussion

Our study provides important insights into the prevalence and implications of having SAM with a disability; 6.4% of children admitted to a SAM treatment ward in Malawi had a clinically obvious disability, the most common being CP. They had a different phenotype compared with those without a disability and were older, less likely to be HIV positive or have oedema; and were more severely malnourished at admission. A key finding of our study is that they were also at significantly greater risk of dying, both during treatment and after discharge: only 11 of the original 60 children were known to have survived until 7 years post-SAM.

The mortality risk is striking. By 7 years post-treatment, children with disabilities have almost seven times greater risk of dying than those without a disability. Mortality for children with two specific types of disabilities, CP and hydrocephalus is particularly high. While life expectancy for both conditions depends on the severity of the condition and co-existing medical concerns, this mortality is nonetheless, reason for further inquiry.

The second key observation is a marked difference in clinically obvious versus screening-tool identified disability. While a structured assessment tool such as Washington Group Questionnaire confirms disabilities identified clinically, many who were not originally identified as having a disability did have ‘difficulties’ at 7 years post-SAM (though the timing of onset of these difficulties is not possible to say from our data).

We know that children with disabilities are more vulnerable to malnutrition for a variety of reasons, including: physical difficulties in feeding or swallowing (eg, among children with CP), receiving less food or less nutritious food at the household level^3 17^ or exclusion from feeding programmes.^18 19^ Our study confirms this increased vulnerability in this population, and these same factors may also make them more likely to die as a consequence of malnutrition. Since ours was an observational trial, it is not possible to definitively infer causality and which came first, especially as the links between malnutrition and disability are bidirectional.^20^ An important outstanding question is how children with disabilities and SAM compare with their peers with similar disabilities but who receive adequate nutrition.

Despite the small sample of survivors with a disability, we found that those in our study admitted with a clinically obvious disability were more stunted, had less catch-up growth and had smaller waist, hip and head circumference than SAM survivors without a disability by 7 years postdischarge. They also had functional deficits, including significantly weaker hand grip strength, and poor school achievement at 7 years postdischarge than those without a clinically obvious disability at admission. These are similar issues seen in SAM survivors compared with controls,^7^ but are even more severe in SAM survivors with disabilities.

Another significant finding is the additional value that a structured disability questionnaire such as the Washington Group tool can provide over and above clinical assessment for disability. Although the Washington Groups questions are not a diagnostic tool, they do provide insight into the presence and severity of physical or learning/behavioural difficulties. We found 100% overlap between those diagnosed with a disability clinically at admission and those with ‘a lot or more’ of any difficulty, using the Washington Group questions. However, there were many children with ‘a lot or more’ physical difficulties that were not identified at original admission (6%), and an even greater number (30%) of children with ‘a lot or more’ learning/behavioural difficulties who were not identified at admission. Some of these difficulties may have been new, acquired postadmission but a more likely explanation is that they were present but missed during non-specific clinical assessment. This highlights the utility of the Washington Group questions and underscores the need for formal assessment and better training on identification of disability within malnutrition care. If a disability is never identified then opportunities to properly support that child and his/her family are correspondingly limited.

Several recent studies relating to the treatment of acute malnutrition have suggested that programmes will be able to more effectively treat children if they focus on identifying the most ‘at-risk’ children, which may not simply be the most severely wasted children. For example, children who are concurrently wasted and stunted are at greater risk than those who are only wasted.^20^ Younger infants, especially those <6 months old, as well as some moderately malnourished children are also likely to be at high risk of death and deterioration.^21 22^ Our results concur with this concept and urge policymakers and practitioners to include disability while considering the future of SAM treatment programmes.

Comparing the Washington Group questions for SAM survivors with controls, we found that sibling controls had few physical and behaviour difficulties, whereas community controls had similar behaviour difficulties to SAM survivors but fewer physical difficulties. The lower prevalence of difficulties reported by caregivers for sibling controls could be due to the relative and direct comparison with the SAM surviving child. It is not possible to know whether the caregivers therefore reported greater relative difficulties in their SAM surviving child, or fewer relative difficulties in their sibling. Our recent study of cognitive function and brain MRI scans for this same group found apparent preservation in gross brain structure in SAM survivors compared with controls, however they did have reduced school achievement and weak evidence of poorer performance on a cognitive function test.^13^ We discussed whether poorer school achievement for SAM survivors could be due to the perception of stunted children, who may appear younger than they are, affecting when they are enrolled in school, when they move up grades and their social interactions with adults.^23 24^ Whether the greater difficulties in SAM survivors are biological or perceived, they have equally important functional implications.

### Limitations

Although many impacts of disabilities were found by this study, we acknowledge that false negative results could be explained by the large survivor bias within our sample. This applies to the group of SAM survivors compared with controls, as well as to SAM survivors with a disability. If the mortality rate of children with SAM and a disability were reduced, and more children survive, even greater long-term morbidity/functional impairments within the population are likely. It was also difficult to achieve accurate measurements for anthropometry and bioelectrical impedance analysis for some children with disabilities resulting in a small and potentially biased sample. We did not use modified processes for conducting anthropometry in this population and we did not compared with specialised growth charts for children with CP.

Another limitation of our study is potential selection bias of our community control group. While the study team employed a systematic random method for selecting a child in the community of the same age and sex as the case child, they were reliant on households volunteering their eligible child to take part, or referring them to eligible neighbours. Children with more severe disabilities may have been less likely to be volunteered to our study team. Also possible is that those with problems were more likely to accept a clinical review (healthy neighbours maybe did not see the point of being assessed by our team of nurses)—this might explain the high rates of reported problems according to Washington Questions in our control group. Reporting bias with regard to comparison between SAM surviving children and their siblings may also be an issue, as already discussed. Lastly, it is important to note that, as an observational trial, it is not possible to determine causality and direction of relationship: whether disability or malnutrition came first.^25^

Finally, data for this cohort were collected using the Washington Group questions prior to development and adoption by a joint UNICEF/Washington Group task force of the Child Functioning Module (CFM). The CFM has two components: a module for children 2–4 years of age composed of 16 questions covering 8 core domains of functioning, and a module for children 5–17 years of age composed of 24 questions covering 12 core domains of functioning. Our findings remain valid, but future research using the CFM may produce more nuanced insight into SAM in children with disabilities.^22^

## Conclusion

Mortality among children with disabilities at 1 and 7 years postdischarge was markedly higher than for non-disabled children admitted with SAM. Disability is second only to HIV as a risk factor for death—yet resources available for these two conditions differ hugely. Underlying disability was prevalent in a population of children with SAM, and those with a disability have even poorer growth, strength and school achievement than other SAM survivors. These results highlight the need for malnutrition prevention, treatment and long-term support for children with disabilities. Interventions to reduce the high postdischarge mortality of children with disabilities is a priority. Also vital is better support to improve development and in turn, school achievement. To better help all SAM survivors to both survive and thrive, SAM programmes, as well as research studies, must become better at identifying and including children with disabilities. The Washington Group questions could be a simple, practical indicator for community programmes to better understand the population of children they are serving. Improving structured assessments of children through tools and better training could also help identify these vulnerable children.
