# Supplementary material for: Long-term outcomes for children with disability and severe acute malnutrition in Malawi
Source: BMJ Glob Health. 2020 Oct 7;5(10):e002613. doi: 10.1136/bmjgh-2020-002613 (PMC7542612; doi:10.1136/bmjgh-2020-002613)
Supplement: Supplementary data [file bmjgh-2020-002613supp001.pdf]

Annex Table 1: Factors associated with disability at admission

	Difference between those with disability and those without (95% CI)	P value	Adjusted difference between those with disability and those without (95% CI)	P value
**Sex (male)#**	0.45 (-0.09, 0.10)	0.10	0.54 (-0.06, 1.13)	0.08
**HIV#**	-1.07 (-1.71, -0.43)	0.001*	-1.08 (-1.73, -0.43)	0.001*
**Oedema at admission#**	-0.25 (-0.80, 0.30)	0.38	-0.82 (-1.50, -0.13)	0.02*
**WHZ at admission**	-0.85 (-1.38, -0.32)	0.002*	-1.31 (-1.81, -0.81	<0.001*
**WAZ at admission**	-0.92 (-1.39, -0.46)	<0.001*	-1.27 (-1.72, -0.82)	<0.001*
**HAZ at admission**	-0.74 (-1.16, -0.31)	0.001*	-0.78 (-1.21, -0.35)	<0.001*

#logistic regression used for categorical outcomes, otherwise linear regression used for continuous outcomes.
Adjusted difference includes= age, sex and HIV status as covariates

Lelijveld N, *et al. BMJ Global Health* 2020; 5:e002613. doi: 10.1136/bmjgh-2020-002613

Annex Table 2: Prevalence of responses to Washington group questions

**Washington Group Disability Questions**		**SAM survivors (n=314)**			**Sibling controls (n=209)**			**Community controls (n=175)**			**All children (698)**		
**Number**	**Type of difficulty**	**At least some difficulty**	**At least a lot of difficulty**	**Can't do at all**	**At least some difficulty**	**At least a lot of difficulty**	**Can't do at all**	**At least some difficulty**	**At least a lot of difficulty**	**Can't do at all**	**At least some difficulty**	**At least a lot of difficulty**	**Can't do at all**
1	Difficulty seeing	17 (5.5%)	5 (1.6%)	0 (0%)	4 (1.9%)	2 (1.0%)	0 (0%)	9 (5.2%)	0 (0%)	0 (0%)	30 (4.3%)	7 (1.0%)	0 (0%)
2	Difficulty hearing	52 (16.6%)	14 (4.5%)	0 (0%)	14 (6.7%)	3 (1.4%)	0 (0%)	22 (12.6%)	2 (1.1%)	0 (0%)	88 (12.6%)	19 (2.7%)	0 (0%)
3	Difficulty walking	13 (4.2%)	10 (3.2%)	6 (1.9%)	4 (1.9%)	2 (1.0%)	0 (0%)	1 (0.6%)	0 (0%)	0 (0%)	18 (2.6%)	12 (1.7%)	6 (0.9%)
4	Difficulty with self-care(feeding/dressing)	24 (7.6%)	11 (3.5%)	5 (1.6%)	13 (6.4%)	1 (0.5%)	0 (0%)	10 (5.7%)	1 (0.6%)	0 (0%)	47 (6.8%)	13 (1.9%)	5 (0.7%)
5	Difficulty understanding	40 (12.7%)	14 (4.5%)	2 (0.6%)	11 (5.3%)	1 (0.5%)	0 (0%)	10 (10.9%)	3 (1.7%)	0 (0%)	70 (10.0%)	18 (2.6%)	2 (0.3%)
6	Difficulty being understood	32 (10.2%)	10 (3.2%)	6 (1.9%)	5 (2.4%)	0 (0%)	0 (0%)	7 (4.0%)	0 (0%)	0 (0%)	44 (6.3%)	10 (1.4%)	6 (0.9%)
7	Difficulty learning	64 (20.5%)	28 (9.0%)	6 (1.9%)	18 (8.6%)	5 (2.4%)	0 (0%)	23 (13.1%)	3 (1.7%)	0 (0%)	105 (15.1%)	36 (5.2%)	6 (0.9%)
8	Difficulty remembering	102 (32.6%)	57 (18.2%)	8 (2.6%)	33 (16.4%)	12 (6.0%)	1 (0.5%)	43 (24.7%)	15 (8.6%)	1 (0.6%)	178 (25.9%)	84 (12.2%)	10 (1.5%)
9	Worry more	74 (23.6%)	7 (2.3%)	7 (2.3%)	42 (20.1%)	1 (0.5%)	1 (0.5%)	43 (24.6%)	7 (4.2%)	7 (4.2%)	159 (23.8%)	15 (2.3%)	15 (2.3%)
10	Difficulty controlling behaviour	60 (19.2%)	33 (10.6%)	5 (1.6%)	25 (12.3%)	6 (2.9%)	0 (0%)	42 (24.3%)	18 (10.4%)	0 (0%)	127 (18.4%)	57 (8.3%)	5 (0.7%)
11	Difficulty completing tasks	77 (24.6%)	25 (8.0%)	6 (1.9%)	24 (11.8%)	6 (3.0%)	0 (0%)	43 (24.6%)	10 (5.7%)	0 (0%)	144 (20.8%)	41 (5.9%)	6 (0.9%)
12	Difficulty with change of routine	61 (19.4%)	29 (9.2%)	4 (1.3%)	26 (12.9%)	12 (6.0%)	0 (0%)	31 (17.7%)	12 (6.9%)	0 (0%)	118 (17.1%)	53 (7.7%)	4 (0.6%)

13	Difficulty getting along with other children	43 (13.7%)	13 (4.2%)	3 (1.0%)	15 (7.4%)	4 (2.0%)	0 (0%)	15 (8.6%)	9 (5.1%)	0 (0%)	73 (10.6%)	26 (3.8%)	3 (0.4%)
14	Difficulty playing	39 (12.4%)	10 (3.2%)	4 (1.3%)	14 (6.7%)	6 (2.9%)	0 (0%)	9 (5.1%)	1 (0.6%)	0 (0%)	62 (8.9%)	17 (2.4%)	4 (0.6%

Annex Table 3: Prevalence of the disability according to Washington Group questions (summated)

Type of disability	SAM survivors (n=314)	Sibling control (n=209)	Community control (n=175)	All children (698)
Physical difficulty (question 1-4)	85 (27.1%)	32 (15.3%)	36 (20.6%)	153 (21.9%)
Learning / Behaviour difficulty (5-14)[#]	208 (66.2%)	109 (52.2%)	125 (71.4%)	442 (63.3%)
Any difficulty	221 (70.4%)	115 (55.0%)	134 (76.6%)	470 (67.3%)
"A lot or more" of physical difficulty	29 (9.2%)	7 (3.4%)	3 (1.7%)	39 (5.6%)
"A lot or more" of learning / behaviour difficulty[#]	102 (32.5%)	36 (17.2%)	54 (30.9%)	192 (27.5%)
"A lot or more" of any difficulty	109 (34.7%)	39 (18.7%)	56 (32.0%)	204 (29.2%)

[#]only includes children who worry more, not worry less

Annex Table 4: Associations between Washington group questions at follow-up and surviving SAM

Washington Group Disability Question Number	Type of difficulty	Sibling controls adjusted OR	P-value	Community controls adjusted OR	P-value
1	Difficulty seeing	0.47 (0.13, 1.69)	0.25	1.50 (0.51, 4.39)	0.46
2	Difficulty hearing	0.44 (0.22, 0.88)	**0.02**	0.75 (0.38, 1.46)	0.40
3	Difficulty walking	0.35 (0.07, 1.70)	0.19	0.16 (0.02, 1.61)	0.12
4	Difficulty with self-care(feeding/dressing)	1.37 (0.62, 3.02)	0.43	1.30 (0.55, 3.07)	0.55
5	Difficulty understanding	0.44 (0.20, 0.94)	**0.03**	0.86 (0.43, 1.75)	0.68
6	Difficulty being understood	0.29 (0.09, 0.87)	**0.03**	0.61 (0.23, 1.62)	0.32
7	Difficulty learning	0.44 (0.24, 0.84)	**0.01**	0.68 (0.36, 1.27)	0.23
8	Difficulty remembering	0.39 (0.24, 1.04)	**<0.0001**	0.64 (0.39, 1.04)	0.07
9	Worry more	0.93 (0.57, 1.49)	0.76	1.00 (0.10, 1.01)	0.52
9	Worry less	1.42 (0.95, 2.13)	0.09	1.03 (0.67, 1.59)	0.90
9	Worry more or less	1.50 (0.93, 2.42)	0.09	1.22 (0.74, 2.01)	0.43
10	Difficulty controlling behaviour	0.57 (0.32, 1.01)	0.05	1.22 (0.72, 2.08)	0.46
11	Difficulty completing tasks	0.42 (0.24, 0.74)	**0.003**	0.97 (0.58, 1.62)	0.91
12	Difficulty with change of routine	0.67 (0.38, 1.18)	0.17	0.81 (0.45, 1.45)	0.48
13	Difficulty getting along with other children	0.59 (0.29, 1.19)	0.14	0.71 (0.34, 1.47)	0.35
14	Difficulty playing	0.64 (0.30, 1.35)	0.24	0.46 (0.19, 1.14)	0.10

Logistic regression adjusted for age, sex and HIV status

Annex Figure 1: Changes in anthropometric z-score since admission for children with and without a clinically obvious disability

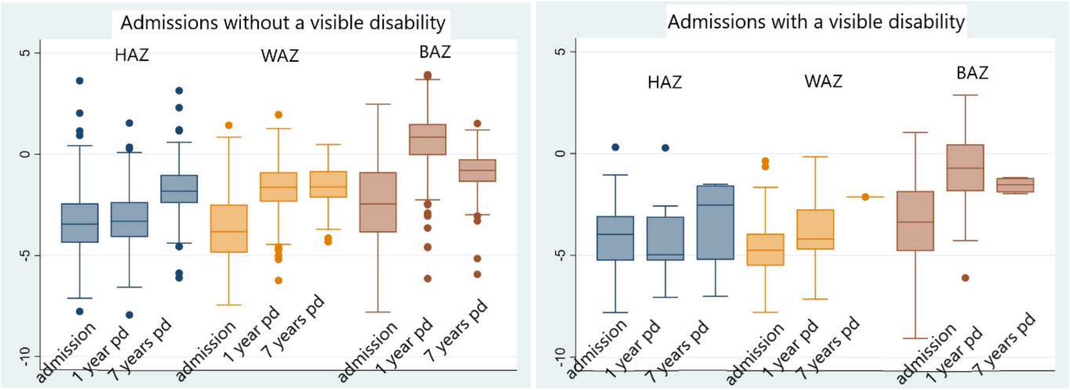

HAZ= height for age z-score; WAZ= weight for age z-score; BAZ= BMI for age z-score. PD= post-discharge
